# Patient influence in home-based reablement for older persons: qualitative research

**DOI:** 10.1186/s12913-017-2715-0

**Published:** 2017-11-15

**Authors:** Aud Moe, Kari Ingstad, Hildfrid V. Brataas

**Affiliations:** 1 Faculty of Health Science, Nord University, Postboks 1490, N-8049 Bodø, Norway; 2 Centre of Care Research, Central Norway, Postboks 1490, Bodø, N-8049 Norway

**Keywords:** Patient influence, Older persons, Home-based reablement, Qualitative research

## Abstract

**Background:**

Reablement services are rehabilitation for older people living at home, being person-centered in information, mapping and the goal-setting conversation. The purpose of this study was to gain knowledge about conversation processes and patient influence in formulating the patients’ goals. There are two research questions: How do conversation theme, structure and processes appear in interactions aiming to decide goals of home-based reablement rehabilitation for the elderly? How professionals’ communication skills do influence on patients' participation in conversation about everyday life and goals of home-based reablement?

**Methods:**

A qualitative field study explored eight cases of naturally occurring conversations between patients and healthcare professionals in a rehabilitation team. Patients were aged 67–90 years old. The reablement team consisted of an occupational therapist, physiotherapist, nurse and care workers. Data was collected by audio recording the conversations. Transcribed text was analyzed for conversational theme and communication patterns as they emerged within main themes.

**Results:**

Patient participation differed with various professional leadership and communication in the information, mapping and goalsetting process. In the data material in its entirety, conversations consisted mainly of three parts where each part dealt with one of the three main topics. The first part was “Introduction to the program.” The main part of the talk was about mapping (“Varying patient participation when discussing everyday life”), while the last part was about goal setting (“Goals of rehabilitation”).

**Conclusions:**

Home-based reablement requires communication skills to encourage user participation, and mapping of resources and needs, leading to the formulation of objectives. Professional health workers must master integrating two intentions: goal-oriented and person-centered communication that requires communication skills and leadership ability in communication, promoting patient influence and goal-setting. Quality of such conversations is complex, and requires the ability to apply integrated knowledge, skills and attitudes appropriate to communication situations.

## Background

Rehabilitation interventions may support elderly patients’ resources. Rehabilitations are planned processes where multiple actors collaborate to provide necessary assistance to the client’s own efforts to achieve the best possible functioning coping ability and participation in social life [1]. The aim is to improve the person’s performance in everyday living [2], and to create health in everyday life [3]. The focus of this study is elderly patients’ influence in conversations with healthcare professionals about performance in everyday living and goals for home-based rehabilitation.

### Home-based reablement for older persons

Different denominations exist about rehabilitation for older people living at home; for example, reablement [4–6]. This is a time-limited home-based rehabilitation for elderly with functional change [7, 8]. Reablement is a service that seems to be under development [9]. Some research has shown uncertain outcomes [5], while other research has shown that reablement has led to a need for reduced home care [10].

Reablement services are described as being person centered, [11] using the client-centered instrument, ‘Canadian Occupational Performance Measure’ (COPM), which is designed to promote user-centered practice [12]. However, Sugavanam et al. [13] found in a review study that patients experienced their roles as indistinct in the goal setting process. A study by Hjelle et al. [8] showed that elderly participation depended on intrinsic motivation interacting with external motivation. Another study revealed that social support and relationships affected participation in home-based reablement [14]. Trusting relationships and good interaction are conditions for the person’s active participation in the discussion about needs, objectives and central issues in a rehabilitation process [15].

### Person centered communication

Interpersonal communication in face-to-face interaction are person-centered in the sense that those communicating deal with each other as individuals with personal qualities and roles [16, 17]. The interaction between healthcare professionals and patients is central to the effective application of patient-centered care [18]. Person-centered communication is humanity and attitude-based, relationship driven, and oriented toward jointly developing dialogue [17]. Communication goals are intentional by relating to what the speaker wants to achieve, and intentionally related to the other person’s thoughts and feelings [19, 20]. The intention of conversations before rehabilitation is unlike typical intentions of communication in health care settings, which is to influence the patient’s health status or state of well-being [21]. Before reablement rehabilitation, the intention is that patients should set rehabilitation goals themselves [22]. Leading the interaction, the professional needs structuring skills as well as informing, explaining and clarifying skills, and open questioning and follow-up skills. The professional may need to reduce vagueness and use active listening skills [16].

### Complexity of goalsetting communication

COPM is a tool in the structuring of conversations, and often used in home-based reablement goal-setting processes. But conversations are complex, and still challenging. Research aiming to better understand the complexity of patients’ self-management in rehabilitation goal setting, highlight the need of health care professionals’ skills, and attitudes to negotiate and decide on goals with patients [23]. A review shows that in stroke rehabilitation, patient-centered goalsetting is minimally adopted [24]. In fact, it is a challenge for professionals to coach patients to set their goals themselves [22].

This challenge points to the need for more knowledge in order to promote quality of patient-centered communication in goal-setting practice. Patients may experience unclear roles, motivators, and relationships when they set their own goals. There is little knowledge of mapping and goal setting conversation in reablement. The purpose of this study was to gain knowledge about conversation processes and patient influence in formulating the patients’ goals, and this lead to two research questions:

- RQ 1. How do conversation themes, structures and processes appear in interactions aiming to decide on goals of home-based reablement rehabilitation for the elderly?

Based on findings under RQ1, we further want to investigate:

- RQ 2. How professionals’ communication skills do influence on patients’ participation in conversation about everyday life and goals of home-based reablement?

## Methods

This qualitative field study was a naturalistic inquiry exploring eight cases of naturally occurring conversations between patients and healthcare professionals in a rehabilitation team [25, 26]. Content analysis is appropriate when gaining knowledge about theme and patterns of patients’ influence in discussions with health professionals [26, 27]. Within-case themes compared were across all eight cases in cross-case analyses [26]. The study assumes a skills perspective on communication in meetings between people [16, 20].

### Informants and recruitment

The head administrator of a home-based reablement rehabilitation project assisted with the provision of information and selection of participants. The sample was information-rich, and a purposeful appropriate selection was used [27, 28].

*The sample* included eight conversations about patients’ performance in everyday living and goals for their home-based restorative rehabilitation. Purposive sampling is a method in which participant selection is based on judgment about who will be most representative and informative [28]. It was desirable that the variation in patient selection reflected the variations of patient situations occurring in ordinary rehabilitation practice. Conversations were chosen consecutively, with up to eight included. The sample with eight cases, considered to provide a reasonable coverage of the conversation phenomenon, given the purpose of the study, and the variation in health conditions, sex, marriage/ single living patients, and professionals conducting conversations [29].

Patients were aged 67–90 years old (mean = 80 years). They lived in their own private homes. The informants had different diagnoses; for example, depression, chronic obstructive pulmonary disease, diabetes, heart/vascular disease, and renal failure, but diagnosis was not the basis for the selection. Health professionals that conducted the talks were included consecutively. The team consisted of an occupational therapist, physiotherapist, nurse and care workers with training in rehabilitation (Table 1).

**Table 1:** Descriptions of the sample

Sex	Age group	Marital status	Partner present	Health workers present
Woman	86–90 years old	widow	no	2
Woman	81–85 years old	widow	no	1
Woman	66–70 years old	married	no	2
Man	71–75 years old	married	no	1
Man	86–90 years old	married	yes	2
Woman	76–80 years old	married	no	2
Woman	66–70 years old	widow	no	2
Man	86–90 years old	married	yes	2

Partner present: yes/ no

Health workers present: one health workers = 1, two health workers present = 2

### Data collection

Researchers distributed audio recorders to health care personnel and instructed on the use of the recorder to the personnel who conducted the conversations.

COPM used by health professionals is a semi-structured interview format and a structured scoring method to document the client’s self-perception of his/her own activity performance and his/her own rehabilitation goals [30]. Data collected were through audio recordings of conversations between patients and health professionals. Some patients had a spouse present. Each conversation included one or two health workers from the rehabilitation team. One health worker led the conversation. Information and mapping the conversation aimed to formulate the patient’s goals for a rehabilitation program that would last for 4–6 weeks. The patients were supposed to decide what goals were right for themselves to cope in everyday life. The talks took place in the patient’s home.

After the conversation, the sound recorder was sent back to the researchers. The recordings then were transcribed into text, word for word, with a selection of who said what.

### Data analysis

Transcribed text was analyzed for conversational theme and for communication patterns as they emerged within main themes [19, 20, 28]. Content analyses were going through four phases, inspired by Vaismoradi [31].

In the *initialization phase* transcribed text was read in its entirety several times to get an overall understanding of the data and what was important in the conversation phenomenon. Thereafter, units of meaning were coded to text close to designations for conversation topics. This process reduced the raw data to text abstractions relevant to the research question. Written notes provided an opportunity for researchers to ask questions about the meaning of the conversations.

The next phase of the analysis, *construction,* emphasized theme and category construction, and proceeding of theme through conversations. Classifying codes in categories was a determination of typology based on similarities in meaning. Codes were mutually exclusive. Category refers to explicit content of text. A theme is more general and abstract and has intellectual and affective content depending on the interpretation of the researcher [31]. Theme settled was through consensus among the three scientists. A summary of topics is provided in Table 2.

**Table 2:** Summary of conversation theme

1 Introduction to the program		2 Varying patient participation when discussing everyday life		3 Goals of rehabilitation	
1A	Information about the program	2A	The patient situation and coping experience	3A	Specification of goals (who, what, how)
1B	Patient’s understanding of the program and mapping	2B	Needs assessment	3B	Satisfaction with the present situation and importance of new rehabilitation goals
1C	Patient motivation to participate in the program	2C	The patient’s context	3C	Summing up, closing
1D	Adaptation of daily life for participation in the program				

Maps were made over the course of the theme of each conversation. Thematically flow of themes through conversations were explored, with emphasis on subtheme of the three themes discussed in the conversation. Table 3 gives an overview of thematic content in raw data of all the conversations.

**Table 3:** Theme-flow through the conversation in reablement

Informant 1
648 expressions ➔
1B – 1A – 1C – 1A – 1D – 1A – 2A – 2A – 2A – 2B – 3A – 1A & B – 2A – 2A – 2B – 3A – 1D – 3A – 2A – 3A – 1C – 2A – 2A – 3A – 2A – 2A – 2A – 2B – 2A – 2C – 2A – 2A – 2A – 3A – 2B – 3A – 3A – 3A – 2B – 1C – 3B – 3B – 3B – 1B – 2B –3B – 2B – 3B – 2B – 3B – 2B – 3B & 1C – 3C –1C
Informant 2
941 expressions ➔
2A – 1A – 2A – 2B – 2A – 2B – 2B – 2A – 2A – 2A – 2A – 2A – 2A – 2C – 2A – 2A – 2A – 2A – 2B – 2A – 2B – 2A – 2B – 2A – 2A – 2A – 2B – 2B – 2A – 3A – 2A – 1C – 2A – 3B – 2A – 3A – 2B – 2A – 3B – 1A – 2A – 1A – 2C – 3B – 2A – 3B – 2A – 2A – 2A – 1D – 3C – 1D – 3C
Informant 3
190 expressions ➔
3A – 3A – 3A – 3A – 3A – 3B – 3B – 3B – 3B – 3B – 1C – 3A – 3B – 3B – 3B – 3B – 3B – 2A – 1C – 3B – 3B – 3C
Informant 4
228 expressions ➔
1B –1A – 2B – 3A – 2B –2A – 2B – 2A – 2B – 3A – 2A – 2B – 3A – 2A – 3A – 2A – 2B – 2A – 2B – 3A – 1C – 2B – 2A – 2B – 2A – 1A – 2A – 1A – 2A – 1A – 2B – 3A – 1C – 2B – 3A – 2A – 3A – 2A – 3A – 1B – 2B – 2A – 2B – 1A – 3B – 2B – 3B – 2A – 2B – 2A – 3A – 2A – 3A – 2A – 3C
Informant 5
469 expressions ➔
1C – 1D – 2B – 1A – 2B – 2A – 2B – 2A – 1D – 3A – 1D – 1A – 3B – 1A – 2B – 1A – 3A – 1A – 1B – 1D – 1A – 1B – 1C – 3A – 1A – 1A – 1C – 2B – 1B – 1A – 1B – 1A – 2A & 2B – 2B – 2A – 2B – 2A – 2B – 2A – 2B – 2A – 2B – 2C – 2B – 2A – 2B – 1B – 1A – 1B – 2A – 2B – 2A – 1C – 2A – 1D – 1C – 1A – 3A – 2B – 3A – 3A – 2C – 2B – 2A – 2C – 2A – 1B – 1A – 1B – 1A – 2A – 1A – 2C – 3A – 2A – 1B – 3C
Informant 6
470 expressions ➔
1A – 2A – 2B – 2B – 2B – 2C – 1A – 2A – 1A – 2A – 3A – 2B – 1A – 1A – 2C – 2C – 2C – 2B – 2A – 2B – 2B – 2A – 3A – 3A – 2A – 3A – 3A – 2A – 1C – 2A – 2B – 2B – 2A – 3A & 2B
Informant 7
397 expressions ➔
1A – 2A – 2A – 2B – 2A – 2B – 2B – 2B – 2B – 2B – 2B – 1A – 2B – 2B & 1C–1A – 2A & 2B – 2B – 2A & 2B – 2B –2A & 2B – 2B & 2C – 2B – 2B – 2B – 2B – 2B – 2B – 2B – 2B – 1A & 2B – 2B – 1A & 1B – 2C – 1A & 3C
Informant 8
316 expressions ➔
1A – 1B – 1C – 1A – 2A – 2B – 2A – 2B – 2A – 2B – 2B – 2A – 2B – 2A – 2B – 2A – 2B – 2A – 2B – 2A – 2B – 2A – 2B – 1C – 2C – 2B – 2B – 2B – 2A – 2B – 1A – 3A – 1C – 3A – 1C – 3A – 1A – 2B – 3B – 3B – 1A – 1B – 1A – 1C – 1D – 2A – 1C – 3C

Once again, all the material was reviewed and highlighted for various means of communication the informants used. Patterns of healthcare and patients’ contributions to the discussions within each main theme were analyzed [19]. In the interpersonal process, goals of each participant are central. Goals can be assertive, relational, or task-oriented, and can be provided by others, be self-selected or be negotiated [16, 20]. Communication goals, and ways to go forward and respond to goals, were marked through all eight conversations. Within each theme, we looked for characteristics of the communication process that were signs of the patients’ influence and active participation. During this part of the analysis, each researcher analyzed parts of the material. Subsequently, the analyses were gone through jointly.

Before the next phase, *rectification*, scientists distanced from data material in a period, in order to strengthen the sensitivity and minimize premature and incomplete process analysis. The results were compared to research literature. This phase was a verification of the process of analysis. We got an overview of topics and communication patterns during the conversation about the various topics.

*Finalization* is the phase with the development of “the story line” that illuminates the research questions [31]. It led to a holistic overview of topics and communication patterns in the information and mapping conversations. Findings provided information of various prerequisites for patients’ influence in the conversations.

The findings constitute the informants’ voices through the processing and analysing of the text. The literature assessment and the discussion provide room for the researchers’ voices with interpretations and an emphasis on the professionals’ skills in order to promote patients’ participation in the goal-setting process. To achieve reliability, parts of the material were analysed by three researchers, then discussed for concurrence. Attention was given to the validity of the analysis throughout the whole process, from the theoretical presuppositions to forming the research questions through interviewing, transcribing, analysing, validating, and reporting [28]. This was to promote the trustworthiness of the study.

### Ethical approval

The project was approved by the Norwegian Social Science Data services (NSD), project number 34297.

Verbal and written information was provided to the patients and health care professionals by the researchers order to ensure informed voluntary participation. In order to ensure confidentiality, consent for the publication of raw data is not provided. Theme-flow of conversations is available; see Table 3. The results are anonymous, and information that emerges will not lead back to identifiable individuals.

## Results

According to RQ 1, Table 3 shows how the theme proceeded through the conversations. There was no common structure for the theme discussed in the talks. The conversation process (RQ 1) and professionals’ communication skills do have an influence on the patients’ participation in conversation (RQ 2). This shows that, for the data material in its entirety, conversations consisted mainly of three parts: information, mapping, and goal-setting. Each part dealt with one of the three main topics. The first and least-weighted part was the theme “Introduction to the program.” The main part of the talk was about mapping (“Varying patient participation when discussing everyday life”), while a somewhat shorter part was about goal-setting, i.e. “Goals of rehabilitation.” The sub-themes are illustrated in Table 2. Described in the following are the processes in which the professionals’ communication skills do influence patients’ participation in conversation about everyday life and the goals of home-based reablement.

### Introduction to the program

Patients often got a very brief introduction about both the conversation purposes and what the rehabilitation would entail. The introduction was characterized by one-way communication, where the staff member would steer the talk and the patient replied briefly and often affirmatively. The staff member opens the door for a few questions when they give information. To a small extent, the patients receive answers to their questions, or ask their own self-set questions. As an example from an introduction sequence, a dialogue where there were two staff members present took place as follows:

Staff member 1: We have received an application from you, stating that you are applying for home nursing, practical assistance in the home. Was it you who ticked this?

Patient: I only signed under it*.*

Staff member 1: It says that you are applying for meal delivery and that I should talk to you about rehabilitation. Is that what you want, this home-based rehabilitation?

Staff member 2: This is a new service we have in the municipality. We're not quite sure if you have received information about this. Then we must arrange for you to get it. It is a new service that started in autumn 2014. And it is basically a team with a physiotherapist, an occupational therapist and home nurses who are engaged in training or rehabilitation in people's homes. It is spread over periods of 4 or 6 weeks, so it's not such a long period. It also means that we work on or help train you for goals that you decide and set for yourself. There can be very many different goals, ranging from helping you to get out of your house to collect your mail, or more advanced things. So, that means that people have very different aims*.*

Patient: I have no problem with that. I drive, I walk and fetch my mail and go for walks. And I go to a physiotherapist*.*

Staff member 1: When we receive an application for assistance, we usually have a survey visit to see what is needed, and whether there is anything we can help you to train for. This is an offer for a service, not something you must have or should have. It is an offer that is new, and today we will talk just a little about what you need and what you might think of*.*

*Patient: Yes.*

This example illustrates that the information about the rehabilitation program was short and disjointed. The staff decided the conversation topics. They never even got comments from the patient; they didn’t ask the right questions. This also was something the staff came back to two or more times during the conversations. Introduction to mapping is largely based on the staffs’ one-way communication; it appears unclear whether the patients understood what the rehabilitation program could mean in their situation, and unclear whether they understood staffs carers’ mapping intentions during the following conversation. It occured talk about patients’ motivation to participate in the program. When motivation seemed low, the employees introduced adaptation of daily life as a topic.

### Varying patient participation when discussing everyday life

The entire conversations were directed by staff in that the staff member introduced the theme. The patients’ influence during the mapping part varied. Patients responded basically to what the staff member said, what to answer ascribed by staff. Often the patients responded briefly and affirmatively or politely with “I see,” while other times the patients responded more completely and self-selected what they themselves felt and meant. When the staff member listened actively and followed up on the patient’s self-selected input, one got the patient’s situation mapped more exactly, which is illustrated in the following example:

Staff member: You have ticked meal delivery, for example*.*

Patient: I think it (cookery) is a little difficult. I have become rather disorganized with making meals. There is frequently no dinner. No, there is not*.*

*Staff member: What do you think is the reason for that?*

Patient: I go to the store and shop and so on, I do that. But, I use a wheeled walker, and one doesn't get so much done then. It means that I only buy bread and things like that. To start to organize dinner from scratch, I don't manage to do*.*

*Staff member: Is it that you are missing items then, or is it that you dislike making the food?*

Patient: It is probably that I dislike getting started with it, I think. I keep getting Fjordland (ready-made food) and such. I do that, but *...*

*Staff member: How do you think this is working out?*

Patient: It is not like homemade. It really isn't. But it's all right, for it just needs to be warmed up. So, it's all right in that way too. I do have the microwave so I can warm it up and all that*.*

Staff member: Is there anything on the physical side that is challenging for you to stand and cook dinner?

*Patient: Yes, there’s that too, yes.*

*Staff member: What is challenging about it then?*

Patient: I have poor balance. But I sit down on the wheeled walker sometimes. But when I am alone then I don't buy things and start making some great dinner, no*.*

Staff member: But, if you already have the items and have decided to do it. Is it okay for you to stand for that the time then and once in a while sit on your wheeled walker?

Patient: Yes, it is, yes. I do sit and relax a little there occasionally. That is good, yes. But, I can't manage to stand all the time*.*

When the staff introduces a sub-topic in a neutral way or asks open questions, the patients describe their situations in their own words and explain what is challenging. As illustrated above, as the conversation continues, the staff members began to ask the more restricted, explicit questions, so that the conversation ends by specifying the patient’s situation, which then lays a foundation for the formulation of goals.

In some conversation sequences the patients took control of what sub-theme to discuss by expressing and conveying in self-chosen terms their own points of view of own functioning and activities they wanted to master, or something they had strong opinions about. This is illustrated in the following dialogue:

Staff member: Now we want to focus on the problems you have. Can you say something about that? I see (from information in your journal) that we have talked with you about the fact that you have no problems with personal hygiene*.*

*Patient: No.*

*Staff member: Do you do everything yourself?*

Patient: It is my goal to walk properly. Last Easter I walked every day for 45 minutes, but I can't do that anymore. It has been hard for me with the kidney problems (began with dialysis treatment one year ago)*.*

The staff member wanted to follow COPM structure, but the patient switched the topic to something he himself thought was important, namely, to be able to walk properly is his goal. This manner of steering the topic from the patients’ perspective is rarely seen in these conversations.

However, using open questions and sub-themes seemed to help the patient participate in the conversation.

### Goals of rehabilitation

The patients’ participation in talking about goal setting was greatly influenced by the patterns of professional leadership and communication. Patients’ goals varied between ascribed by the staff, self-set, or mapped after patient-staff negotiations.

The goals which are communicated can be attributed, which means that someone else determines the goals without very much contribution from the patient. Goals were ascribed for the patient from staff and sometimes from relatives who were present during the mapping conversation. An example to illustrate:

Staff member: Yes, it might be best for your diabetes for you not to have four bread meals each day.

*Patient: Yes, that’s right.*

*Staff member: You should have a dinner meal.*

*Patient: Yes, that’s absolutely right.*

Some of the patients were passive and seemed not very motivated for the rehabilitation process. They engaged very little in the goal-setting process, maybe because next of kin and staff ascribed goals for the patient.

When the patients communicated in a self-set way, they themselves chose which areas the rehabilitation process should concentrate on. A patient who during the mapping part explained that one of her feet has been so weak that she has problems going to the toilet on her own, took the initiative herself to set a goal for this area:

*Patient: If I had only been better on this foot, then I think that I could manage all right.*

*Staff member: Yes?*

*Patient: I will be eighty-five years old in January, but I don’t feel that old.*

Staff member: No, then I will write that as your goal, to increase the strength in your leg so that you can continue to get around and go to the bathroom on your own*.*

The staff member listened to what the patient said, and thereafter helped to identify a goal based on the challenges the patient felt she had.

The goals for rehabilitation sometimes come after negotiations between the patient and staff member. An example:

*Staff member: Shall we set a goal about going out?*

*Patient: But I can’t go alone.*

Staff member: No, so you will go together with us. Shall we decide how many 100 meters you will walk then?

*Patient: Well, it’s difficult for me to sit here today and decide that.*

Staff member: Yes, but there are any places that would be nice to walk to, perhaps some road or another, so we can set up a goal that you walk to either here or there?

*Patient’s wife: Previously you have walked to the playground.*

*Staff member: It is far then, to the playground?*

*Patient: 5–6 min each way.*

*Staff member: About two hundred metres then?*

*Patient’s wife: No longer than that, no.*

*Patient: No probably not.*

*Staff member: So, the return trip is about 300 m?*

*Patient’s wife: Yes. That’s about right.*

*Staff member: Now I’ll write that down.*

Professionals who were listening to patients and leading the conversation in a way meant to encourage patients’ self-set or negotiated goal-setting seem to promote the patients‘influence on the clarifying of rehabilitation goals. The results show that, to a varying extent, the patients contributed self-determination to the formulation of goals within rehabilitation. Sometimes the process led to a goal ascribed to by the staff; sometimes it was patient–staff negotiated, and sometimes it was self-set.

## Discussion

Reablement is intensive, time-limited, goal-oriented, holistic and person-centered [7]. The aim is to improve the person’s performance in everyday living [32], and to create health in a continuous process in the context of everyday life [3]. Use of COPM may enable patients to feel like active participants in the process formulating their own rehabilitation goals [33, 34]. Active goal setting processes seemed developed by clarifying active participation anchored in the patient’s everyday life.

Patient participation differed with varying professional leadership and communication in the information, mapping and goalsetting process. The proposed process was active listening to strengthen the patient’s influence through interaction between the patient and health professional. This could lead to patient-staff negotiations or “self-set goals” that contributed to the patient’s goals. Challenges in the process were sometimes limited patient involvement, which led to ascribed goals formulated by the health worker.

During the introduction phase induction skills set by professionals is of importance for the patient’s involvement in the conversation [16]. Talks provide the basis for the individual rehabilitation program [35] where person-centered communication is oriented toward developing dialogue [17]. Person-centered, the staff develop a clear picture of how patients make sense of what is happening [19]. In this study the rehabilitation program was a new way of thinking about care, where the aim was that patients increasingly became able to master everyday life [30]. Before conversing, the intention is unknown for patients. Thus, the introduction is an important basis for shared responsibility of the development of patients’ rehabilitation goals.

In this study, health workers were sometimes task oriented, others focused on motivating and supporting the elderly. The staff members mainly focused on patients’ everyday life and coping, while a shorter closing part of conversations revolved around the specifying of rehabilitation goals and goal importance. Trappes-Lomax et al. [36] found that the role of staff was of importance being a motivator in the rehabilitation process. Being a motivator is an important part of the role of health workers in helping the patient to decide reachable goal of rehabilitation. The driving force in the process is intrinsic motivation based on a person’s willpower and responsibility. Extrinsic motivation is expressed by being in one’s home environment in co-operation with the reablement team who encouraged and supported the performing of everyday activities [8].

A previous study showed that patients experienced their role as indistinct with differing ideas about goals and achievement for patients and health professionals [13]. Patients’ active participation in the conversations mainly varied with tactics or ways of professional leadership and communication skills used during conversations in this study. When the personnel displayed active listening skills and allowed for patient participation in interactions, this led to patient-staff negotiations and clarification of rehabilitation goals. Sometimes patients’ self-set communication goals contributed to clarification of the patient’s rehabilitation goals. More often the staff limited and controlled patient participation when patients responded to ascribed goals, formulated by the health worker. Findings highlight that patients’ share in communication should be taken more into account than what is found, also in another study of nursing [21].

Unstructured informing managed by the health care personnel points to the need to ensure patient participation based on mutual understanding ensured through dialogue and active listening [20]. The introduction can create the basis for person-centered individualized and respectful care [37]. As the interactions between the elderly and the health care workers improve, the mapping process could contribute to confidence. A study by Randström et al. [38] showed that a prerequisite for participation was mutual relationship with teammates. Factors of importance were respect, being listened to and being supported by staff. This study shows that in various ways and to widely varying degrees, the patients affect the goal clarification process, and we see patients’ participation in communication related to communication skills of the personnel that Hargie [20] categorizes as skills of Self set goals, Collaborative goals and Ascribed goals.

Research has shown that older people experienced that the objectives in the information phase of everyday life rehabilitation were not rooted in the elderly’s life [39]. Trusting relationships may promote the patient’s active participation in the discussion, thus becoming central in goal setting before rehabilitation processes [15, 40]. Content and communication goals are interwoven. There was some encouragement for patients’ self-setting of goals. It occurred when employees asked open questions, and also during a few conversation sequences where patients took control of the conversation and communicated their own views. Focusing, probing, paraphrasing, clarifying, confronting, exploring, summarizing and closing are useful communication techniques [41].

Rehabilitation goals could come forward from negotiations between patient and employee, sometimes also in negotiation with relatives. Professionals’ use of active listening and follow up of patients’ expressions of thoughts and feelings may empower patients’ self-determination of rehabilitation goals [12, 15, 19]. Dialogue where the health staff asked both open and clarifying questions and showed skills in active listening motivated patient participation. External motivation can be expressed as strengthened by cooperation with a reablement team who support the elderly to regain confidence in performing daily activities [8].

A range of communication skills employed are in interactions [16, 20]. In line with other research [22–24], findings of varying autonomous involvement of patients, and some extent of person-centered and individualized goal setting processes, highlight the need of professionals’ communication skills to introduce, negotiate and decide about patients’ rehabilitation needs and goals. There is a need of more research on conversation structuring and health care workers use of communication skills that can promote active dialogue to ensure an offer based on the patient’s own goals for what is meaningful in life.

### Limitations

The method used was appropriate to illuminate research questions. The sample consisted of natural conversations between patients and health workers, and provided rich information. The study was limited to a municipality, but with participants from various districts of the municipality. Employees who participated worked in an interdisciplinary team represented by different professional groups and was a strength of the study. Qualitative research does not only study a few sites or individuals but also collects extensive detail about individuals studied. The intent is not to generalize the information but to elucidate the particular, the specific. The key issue is to generate enough in-depth data that can illuminate the dimensions of the phenomenon under study [28].

Video recording and participatory observation considered as alternative data collection methods, but considered more visible to the participants, and therefore more disturbing than a small recorder in natural conversations. Therefore, audio recordings were chosen in the data collection. In some conversations, one, sometimes two health workers participated. In two of the conversations, relatives also participated. This inequality in conversations bring about variation in data from naturally occurring practice. The needs for the number of participants were different in the conversations due to the varying health statuses of the elderly and the need for assistance from one or more health workers.

## Conclusions

This study focuses on information and mapping conversations in reablement, not the result of the reablement offer. Information and mapping provide the basis for the reablement result in a start-up phase of the offer. In this phase, the result of reablement is currently unknown. Successful rehabilitation requires an effective mapping process and professionals’ excellent communication skills to encourage user participation and mapping of resources and needs leading to the formulation of objectives. Professional health care workers must master merging two integrated intentions: goal-oriented and person-centred communication, which requires communication skills and leadership ability in communication that promotes patient influence and goal-setting.

The study has significance for education and practice with respect to emphasizing competence in person-centered communication. Competence as a prerequisite for quality of such conversations is complex, and requires the ability to apply integrated knowledge, skills and attitudes appropriate in communication situations.

The study points to the need for more knowledge about dialogue where employees influence patients in order to motivate patients for home-based reablement. The study show knowledge of the importance of developing and training expertise, providing person-centered quality of goal setting practice. More knowledge needed is about the appropriate balancing of self-determination and academically grounded external motivation for everyday rehabilitation, this taking the patient perspective into account.

## Abbreviations

COPM: Canadian Occupational Performance Measure
RQ: Research question
